# Nevirapine and tuberculosis predict first-line treatment failure in HIV patients in Indonesia: Case-control study

**DOI:** 10.1016/j.amsu.2020.10.005

**Published:** 2020-10-15

**Authors:** Yanri Wijayanti Subronto, Nur Aini Kusmayanti, Albarisa Shobry Abdalla, Prenaly Dwisthi Sattwika

**Affiliations:** a Division of Tropical Medicine and Infectious Diseases, Department of Internal Medicine, Faculty of Medicine, Public Health, and Nursing, Universitas Gadjah Mada/Dr. Sardjito General Hospital, Yogyakarta, 55281, Indonesia; b Center for Tropical Medicine, Faculty of Medicine, Public Health, and Nursing, Universitas Gadjah Mada, Yogyakarta, 55281, Indonesia; c Department of Internal Medicine, Faculty of Medicine, Public Health, and Nursing, Universitas Gadjah Mada, Yogyakarta, 55281, Indonesia

**Keywords:** ART treatment failure, HIV, Indonesia, Nevirapine, Predicting factors, Tuberculosis

## Abstract

**Introduction:**

Indonesia antiretroviral therapy guideline suggests the use of Non-Nucleoside Reverse Transcriptase Inhibitor (NNRTI)-based regiments as first line of HIV treatment and Protease Inhibitor to replace NNRTI when treatment failure occurred. This case-control study was aimed to study factors predicting first-line ART treatment failure among HIV positive patients aged >15 years, non-pregnant, and registered in our institution, Indonesia.

**Methods:**

Diagnosis of HIV treatment failure was made according to the standard WHO criteria. Demographic and outcome variables were recorded. The association between variables were analyzed by Chi-square test with odds ratios (OR) and 95% confidence intervals (95% CI), followed by multivariate analysis using logistic regression test.

**Results:**

Twenty-six index cases and 26 age- and sex-matched control cases were included in the study with a mean age of 32.27 ± 8.7 years and 32.88 ± 8.15 years, respectively. Median time for switching to second-line (Lopinavir/ritonavir, LPV/r) was 46.32 ± 30.21 months. Patients presented with tuberculosis and treated by nevirapine as the first-line treatment were 26.6-folds (95% CI: 2.41–293.81, *p* = 0.007) and 6.7–folds (95% CI: 1.56–28.45, *p* = 0.011) higher risk for treatment failure, respectively.

**Conclusions:**

The presence of tuberculosis and the use of nevirapine in first-line treatment were strong predictors for first-line ARV treatment failure, suggesting for closer clinical monitoring for patients with those conditions. A further and larger prospective cohort study is needed to confirm the findings in this study.

## Highlights

• Tuberculosis and the use of nevirapine are significant predictors for ARV treatment failure
• Closer clinical monitoring for patients with TB-HIV coinfection is necessary
• Consideration of not using Nevirapine to initiate antiretroviral therapy for patients with HIV who accompanied by TB co-infection

## 1 Introduction

Indonesia is one of the countries with the fastest-growing numbers of HIV infections with approximately 30–40 thousand new cases annually. Until the end of 2018, it is noted that there were approximately 750,000 HIV infected individuals with 103,000 patients were currently on antiretroviral therapy (ART) [1]. According to the 2018 UNAIDS data, Indonesia contributes 18% to the new HIV infection among the Asia Pacific nation, ranked third after India and China. In terms of AIDS-related death, 23% occurred in Indonesia which was ranked second following India [2].

National antiretroviral therapy has started since 2004 using 2 NRTIs (nucleoside reverse transcriptase inhibitor) and 1 NNRTI (non-nucleoside reverse transcriptase inhibitor), namely Zidovudine + Lamivudine and Nevirapine. The national guideline also recommends the use of protease inhibitors (PI) to replace the NNRTI when the first-line treatment failed to maintain clinical, immunological, and or virologic conditions. PI is also given when there is a contraindication or occurrence of severe side effects to NNRTI, such as severe maculopapular rash or neurological symptoms.

Several studies showed that older age, male, severe malnutrition, anemia, low baseline CD4 counts, advanced baseline WHO clinical stage, longer use of ART use, and negative change in body weight were predictors of treatment failure of first-line treatment [3]. Another study in Tennessee showed that black race, female, and younger age were risk factors for discontinuing PI, while black race, younger age, and disability-based enrollment were predictors for discontinuing NNRTI [4]. From our national data, out of 103,000 patients on therapy, 3.2% were on second-line therapy, but little is known about factors predicting first-line ARV treatment failure that lead to the use of PI-based regiment. As treatment failure influenced by many factors, defining predicting factors in each population is thus important.

This case-control study aimed to determine the predictors of first-line treatment failure leading to the use of Lopinavir/ritonavir therapy in patients receiving ART in Dr. Sardjito Hospital, Yogyakarta, Indonesia.

## 2 Materials and method

### 2.1 Study design

This study was a retrospective study using case-control design. Our hospital is one of the main referral hospitals in Indonesia serving patients from the Yogyakarta Special Region and the southern part of Central Java Province. Antiretroviral Therapy (ART) has started 2004 at Edelweis Clinic and currently treating more than 800 patients.

Indonesia follows WHO Guideline on ART for adults and adolescence, *i.e.* using 2 NRTIs and 1 NNRTI as initial therapy or so-called first-line therapy and 2 NRTIs and PI as the second line of treatment [1]. Until 2014, the NRTI used for treatment were mainly Zidovudine (AZT/ZDV), lamivudine (3 TC), whereas the NNRTI drugs were mainly Nevirapine (NVP) and Efavirenz (EFV). Since 2014, Zidovudine was not anymore the main drug of choice and replaced by Tenofovir (TDF). Indonesia then started to use the Fixed-dose combination (FDC) containing Tenofovir + Lamivudine/Emtricitabine + Efavirenz. The second-line regimen was defined as a boosted Protease inhibitor (PI)-based regimen replacing the NNRTI drug in the first line regiment. The most available PI in Indonesia is Lopinavir/ritonavir (LPV/r) and was only available since 2010.

### 2.2 Study subjects

Adult patients ≥15 years old receiving antiretroviral therapy in our hospital between January 2005 and December 2018 were included in this study. From the HIV registry, there were 2712 HIV positive patients who have ever entered the service, with 2396 has ever initiated ART, and 1064 were actively being treated with 959 patients remained in the first-line therapy while 47 on second-line therapy. There were 26 patients meeting the inclusion criteria.

Treatment failure was defined according to the WHO guideline, either clinical, immunological, and or virological failure.

For the study participants, those who were registered using second-line treatment by the end of 2018 were included in the study if they started the first-line therapy at our hospital and were on second-line therapy due to treatment failure of first-line therapy. Pregnant women, patients receiving Lopinavir/ritonavir (LPV/r) as post-exposure prophylaxis, those use PI due to side effects of NNRTI drugs, or those receiving initial ART from another health facilities were excluded from the study. Patients receiving LPV/r as a second line of ART were recorded as case, and those with age- and sex-matched were put in the control group with the ratio of 1:1.

Ethical clearance from the Medical and Health Research Ethics Committee (MHREC) of the Faculty of Medicine, Public Health, and Nursing, Universitas Gadjah Mada/Dr. Sardjito Hospital, states that the research protocol meets the ethical principle outlined in the Declaration of Helsinki 2013 (Ethical approval number KE/0947/08/2018).

This study was registered at the Research Registry (UIN: 5866) and reported in line with the STROCSS 2019 criteria [5].

### 2.3 Data collection

Data was collected, with permission, from the data records of the Medical Record Department, Pharmacy Department, and Edelweis (HIV) Clinic, all located in our hospital. We extracted sociodemographic and clinical data of the patients from their medical records according to standardized data extraction form. The data collection was designed to capture information on age, sex, marital status, occupation, substance use, body weight, body height, CD4 count, viral load, WHO clinical staging, tuberculosis co-infection, ART treatment, the use of Cotrimoxazole prophylaxis, and the level of adherence. Adherence is defined as have more than 90% of pill intake and monthly ARV pick up before the date of changing medication from first-line to PI regimen ART.

### 2.4 Statistical analysis

Data were then coded, entered, cleaned, and analyzed using STATA 16. Sociodemographic and clinical data were presented in simple descriptive statistics including mean ± standard deviations for continuous data and absolute number with percentage for dichotomous data. Factors related to treatment failure was ascertained by comparing variables between the case and control group using a chi-square test for categorical data and Student t-test for continuous variables. Sociodemographic and clinical data of the patients were subjected to univariate analysis. Factors with a p-value of <0.05 at univariate analysis were included in multivariate analysis. Logistic regression was performed to determine factors that may independently predict treatment failure.

## 3 Results

### 3.1 Characteristics at baseline

There were 26 index cases and 26 age- and sex-matched patients in the control group. The median age of those in the case and control group were of 32.27 ± 8.7 years and 32.88 ± 8.15 years, respectively. The median time for switching to the second-line was 46.32 ± 30.21 months. As many as 18 (69%) of the case group were recorded as non-adhere to the first-line therapy but was similar to the control group. Male patients (n = 34) constitute 65% of total patients in the case and control groups. Majority of people in case and control group were single (50% in case group and 50% in control group), has steady work (69% in case group and 58% in control group), and come from heterosexual population (50% in case group and 54% in control group) (Table 1). There were no different social demographic characteristics between case and control group.

**Table 1:** A comparison of the social demographic and clinical characteristics between the case and the control groups at the initiation of ART.

Characteristics	Case (N = 26) n (%)	Control (N = 26) n (%)
Age, mean ± SD (in years old)	32.27 ± 8.7	32.88 ± 8.15
Sex		
Male	17 (65)	17 (65)
Female	9 (35)	9 (35)
Marital status		
Single	13 (50)	13 (50)
Married	10 (38)	11 (42)
Widow/widower Not mentioned	3 [12] 0 (0)	1 [4] 1 [4]
Occupation		
Not working	6 (23)	9 (34)
Steady work	18 (69)	15 (58)
Not mentioned	2 [8]	2 [8]
Risk factor group		
MSM	9 (35)	7 (27)
Heterosexual	13 (50)	14 (54)
PWID^a^	4 [15]	2 [8]
Not mentioned	0 (0)	3 [11]
CD4 count, median ± IQR (cells/μL)	43 ± 110	173 ± 176
HIV clinical stage		
I	3 [11]	7 (27)
II	9 (35)	10 (38)
III	7 (27)	4 [15]
IV	5 (19)	1 [4]
Not mentioned	2 [8]	4 [15]
The presence of tuberculosis		
No	18 (69)	25 (96)
Yes	8 (31)	1 [4]
Tuberculosis plus other co-morbidities		
No	22 (85)	26 (100)
Yes	4 [15]	0 (0)
NNRTI as the first-line drug		
Efavirenz	8 (31)	15 (58)
Nevirapine	18 (69)	11 (42)
Cotrimoxazole prophylaxis		
Yes	13 (50)	5 (19)
No	13 (50)	21 (81)
Adherence before switching Adhere Not adhere	8 (31) 18 (69)	8 (31) 18 (69)

a PWID – people who inject drugs.

At baseline, clinical condition of patients in control group were better than in the case group. Median CD4 cell counts in control group (172 ± 176 cells/mm^3^) was higher than in the case group (43 ± 110 cells/mm^3^). Percentage who were in HIV stage 1 and 2 in control group is higher (65%) than in the case group (46%). Only 4% were suffering from tuberculosis in the control group compared to 31% in the case group, and there was no patient with tuberculosis plus other co-morbidities in control group, but 4 patients (15%) in the case group (Table 1).

### 3.2 Risk factors for switching to second-line regiment

The median time for switching from first to second-line regiment (Lopinavir/ritonavir, LPV/r) was 46.32 ± 30.21 months. Age and sex were matched and risk factors for switching from NNRTI-based to PI-based regimens were analyzed (Table 2). Patients presented with tuberculosis were 26.60-times (95% CI: 2.41–293.81, *p* = 0.007) at higher risks for treatment failure. The use of NVP as first-line treatment was 6.66–times (95% CI: 1.56–28.45, *p* = 0.011) higher risk of developing treatment failure.

**Table 2:** Risk factors for switching to LPV/r in the cohort at multivariate analysis.

Variables	Bivariate Analysis OR (95% CI)	p-value	Multivariate Analysis OR (95% CI)	p-value
Age	0.99 (0.93–1.05)	0.789		
Sex				
Male	1	1.000		
Female	1.00 (0.32–3.13)			
Marital status				
Single	1	0.895		
Married	3.00 (0.28–32.75)			
Widow/widower	0.99 (0.29–2.87)			
Occupation				
Not working	1	0.353		
Steady work	1.80 (0.52–6.22)			
CD4 count	0.99 (0.98–1.00)	0.084		
HIV clinical stage	2.16 (1.07–4.36)	0.032^a^		
Presence of Tuberculosis				
No	1	0.029^a^	1	0.007^a^
Yes	11.11 (1.27–96.86)		26.60 (2.41–293.81)	
Tuberculosis plus other comorbidities				
No	1	*omitted*		
Yes	1			
NNRTI as the first-line drug				
Efavirenz	1	0.054^a^	1	0.011^a^
Nevirapine	3.07 (0.98–9.59)^a^		6.66 (1.56–28.45)	
Cotrimoxazole prophylaxis				
No	1	0.024^a^		
Yes	4.19 (1.21–14.54)			
Adherence before switching				
Adhere Not adhere	1 1.01 (0.99–1.03)	0.168		

a Threshold p-value<0.25 **significant at p-value <0.05.

## 4 Discussion

Our study found that the presence of tuberculosis and the use of Nevirapine (NVP) as first-line antiretroviral therapy significantly predict the switch to second-line regimens. To the best of our knowledge, our study is the first report from Indonesia that implicating the presence of tuberculosis and the use of NVP as strong predictors for the treatment failure. There were no socio demographic characteristics associated with treatment switching. The presence of tuberculosis infection as the predictor for treatment failure was also reported by a study performed in Ethiopia. This multicenter cohort study across countrywide recruiting HIV-infected patients aged ≥14 years old revealed that the rates of treatment failure were 23.3% and 33.9% at 6 and 12 months with risk factors for treatment failure were presence of tuberculosis, presence of opportunistic infection, low CD4 cell count of <50 cells/μL, and high viral load of >5 log10 copies/mL [6].

Concomitant tuberculosis treatment could induce the metabolism of nevirapine, thus reducing its plasma concentration from the third weeks to the end of tuberculosis therapy [7,8]. Low nevirapine plasma concentration led to lower adherence and virological failure [9]. In addition, TB co-treatment impose patient on ART with large pill burden that may impair adherence [10].CD4 cells count are used as the criteria of immunological failure of ARV treatment and the decision to switch ART regimens. The CD4^+^ cells role in both HIV and Tuberculosis infection are seen as fundamental. A systematic review acknowledged the decline in CD4 cell count among HIV positive adults (aged ≥15 years) not receiving ART as a strong risk factor for incident TB [11].

Tsegaya in 2016 reported the risk factors for developing second-line ART treatment failure in adult HIV-infected patients in Ethiopia which revealed that a high rate of failure was found in the first 2 years of ART therapy [3]. In our study, the median time for switching to the second-line treatment was 46.32 ± 30.21 months, but we did not perform survival time analysis. It should be noted, however, that Viral Load machine was only available several years after ART service, there may be possibility that virological failure was already present earlier but unnoticed. A retrospective cohort study in HIV infected children (≤15 years) receiving the first-line antiretroviral therapy at a Tertiary Care Hospital in Ethiopia showed that among 318 children enrolled, the prevalence of treatment failure was 22.6% with 51.4% only immunological failure, 8.3% only virological failure, and 33.3% both failure. The mean time for ARV switching was 12.67 ± 4.96 weeks [12].

In our study, patients with Nevirapine (NVP) as first-line ART were 7.8 times as likely to fail compared to those initiated with Efavirenz (EFV). A retrospective cohort study conducted to children age 6 months up to 18 years old in Kampala, Uganda, also showed treatment failure was related to the use of an NVP-based regimen, in addition to less adherence to ART, and prior use of single-dose NVP [13]. A study in Thailand that concludes children who received a Nevirapine -based regimen had more chance to develop virologic failure than those on Efafirenz-based regimen showed that almost all study subject had NNRTI resistance [14]. Race, in this case, Asian population, had a significantly higher predisposition to develop nevirapine associated rash [15]. A study in Uganda elucidated that low NNRTI plasma concentrations were associated with increased HIV RNA levels (P = 0.02). This study also found that presentation of general symptoms, less body weight compared to baseline at the time of ART initiation, tuberculosis diagnosis during ART, poor adherence, and low NNRTI plasma concentration were associated with virological failure [7].

Our study found that presence of Tuberculosis co-infection alone, but not Tuberculosis plus other opportunistic infections, as predictors of first-line treatment failure. A retrospective 5-years cohort conducted in India, however, revealed that the presence of opportunistic infection, lower BMI, and low CD4 count at ART initiation were predictors of first-line ART failure with a failure rate of 7.69% and incidence of first-line ART failure 2.09 per 1000 person-years [16]. At the start of the first-line treatment, 46% (n = 12) of the case group and 19% (n = 5) of the control group were at WHO clinical stage III or IV. At switch period to second-line ART, a lower CD4 count of <100 cells/mm3 and a WHO clinical stage IV were associated with treatment failure, while weight gain was protective towards second-line treatment failure [3]. Treatment failure among children population were associated with a low CD4 count of <50 cells/mm3 at baseline, WHO clinical stage III-IV at ART initiation, not having both parents, negative serology of caretaker, ART initiation at 11 months or younger [12,17].

In a high TB-burden country, the result of this study could be used to provide information on the rationale for wiser administration of TB drugs and choice of ART. Instead of being the alternative treatment, our study suggest that Nevirapine still can be the drug of choice if all other first-line ART treatment were contraindicated.

There are several limitations of our study, including small number of case and controls, and only involved one center in Indonesia, that might not represent the whole country.

## 5 Conclusions

Our case-control study is the first to report predicting factors of treatment failure of the first-line ART in Indonesia. Presence of tuberculosis and the use of nevirapine as the first-line treatment were strong predictors for ARV treatment failure, suggesting for closer clinical monitoring for patients with TB-HIV coinfection and wiser drug selection to initiate antiretroviral therapy. A further and larger prospective cohort study is needed to confirm the findings in this study.

## Consent

Written informed consent was obtained from the patients for join the study. A copy of the written consent is available for review by the Editor-in-Chief of this journal on request.

## Provenance and peer review

Not commissioned, externally peer reviewed.

## Sources of funding

This study is funded by the Universitas Gadjah Mada Research and Publication Quality Improvement Program.

## Ethical approval

Ethical clearance from the Medical and Health Research Ethics Committee (MHREC) of the Faculty of Medicine, Public Health, and Nursing, Universitas Gadjah Mada/Dr. Sardjito Hospital, states that the research protocol meets the ethical principle outlined in the Declaration of Helsinki 2013 (Ethical approval number KE/0947/08/2018).

## Author contribution

YWS, NAK, ASA, and PDS brainstormed this study.

NAK,ASA,PDS prepared the manuscript draft and YWS critically revised the manuscript for important intellectual content.

NAK,ASA,PDS and YWS facilitated all project-related tasks. All authors read and approved the final manuscript.

## Registration of research studies

Name of the registry: Research Registry.

Unique Identifying number or registration ID: 5866.

Hyperlink to your specific registration (must be publicly accessible and will be checked): https://www.researchregistry.com/browse-the-registry#home/registrationdetails/5f28f38504f3590017191696/.

## Guarantor

Yanri Wijayanti Subronto.

## Declaration of competing interest

The authors declared no potential conflicts of interest.
